# Identification of differentially expressed microRNAs in primary esophageal achalasia by next-generation sequencing

**DOI:** 10.3906/biy-2101-61

**Published:** 2021-06-23

**Authors:** Mahin GHOLIPOUR, Javad MIKAELI, Seyed Javad MOWLA, Mohammad Reza BAKHTIARIZADEH, Marie SAGHAEIAN JAZI, Naeme JAVID, Narges FAZLOLLAHI, Masoud KHOSHNIA, Naser BEHNAMPOUR, Abdolvahab MORADI

**Affiliations:** 1 Golestan Research Center of Gastroenterology and Hepatology, Golestan University of Medical Sciences, Gorgan Iran; 2 Autoimmune and Motility Disorders Research Center, Digestive Diseases Research Institute, Tehran University of Medical Sciences, Tehran Iran; 3 Department of Genetics, Faculty of Biological Sciences, Tarbiat Modares University, Tehran Iran; 4 Department of Animal and Poultry Science, College of Aburaihan, University of Tehran, Tehran Iran; 5 Metabolic Disorders Research Center, Golestan University of Medical Sciences, Gorgan Iran; 6 Department of Microbiology, Faculty of Medicine, Golestan University of Medical Sciences, Gorgan Iran; 7 Department of Biostatistics, Faculty of Health, Golestan University of Medical Sciences, Gorgan Iran

**Keywords:** Achalasia, microRNA, next-generation sequencing, expression profiling, bioinformatics

## Abstract

Molecular knowledge regarding the primary esophageal achalasia is essential for the early diagnosis and treatment of this neurodegenerative motility disorder. Therefore, there is a need to find the main microRNAs (miRNAs) contributing to the mechanisms of achalasia. This study was conducted to determine some patterns of deregulated miRNAs in achalasia. This case-control study was performed on 52 patients with achalasia and 50 nonachalasia controls. The miRNA expression profiling was conducted on the esophageal tissue samples using the next-generation sequencing (NGS). Differential expression of miRNAs was analyzed by the edgeR software. The selected dysregulated miRNAs were additionally confirmed using the quantitative reverse transcription polymerase chain reaction (qRT-PCR). Fifteen miRNAs were identified that were significantly altered in the tissues of the patients with achalasia. Among them, three miRNAs including miR-133a-5p, miR-143-3p, and miR-6507-5p were upregulated. Also, six miRNAs including miR-215-5p, miR-216a-5p, miR-216b-5p, miR-217, miR-7641 and miR-194-5p were downregulated significantly. The predicted targets for the dysregulated miRNAs showed significant disease-associated pathways like neuronal cell apoptosis, neuromuscular balance, nerve growth factor signaling, and immune response regulation. Further analysis using qRT-PCR showed significant down-regulation of hsa-miR-217 (p-value = 0.004) in achalasia tissue. Our results may serve as a basis for more future functional studies to investigate the role of candidate miRNAs in the etiology of achalasia and their application in the diagnosis and probably treatment of the disease.

## 1. Introduction

Achalasia is a chronic neurogenic esophageal motility disorder featured by impaired lower esophageal sphincter (LES) laxity and disturbed peristalsis (Triadafilopoulos et al., 2012). Its symptoms include progressive swallowing disorder, regurgitation, esophageal chest pain, aspiration, and eventually malnutrition (Sadowski et al., 2010). According to a population-based study, achalasia prevalence is more than 10/100,000, with a steady increasing trend from 2.5/100,000 in 1996 to 10.8/100,000 in 2007 (Sadowski et al., 2010). Survival of the patients with achalasia is significantly less than age-sex matched healthy people (Sadowski et al., 2010). Most patients underwent late diagnosis and ineffective treatment due to nonspecific symptoms of the disease and the absence of noninvasive diagnostic tests (Farrokhi and Vaezi, 2007).

The pathophysiology of achalasia is based on selective loss of inhibitory neurons in the myenteric network, which can interfere with the coordination of esophageal peristalsis and LES relaxation during swallowing (Ghoshal et al., 2012). Decreased levels of the nitric oxide synthase (NOS) and vasoactive intestinal polypeptide (VIP) as inhibitory neurotransmitters in the myenteric plexus disrupt esophageal neuromuscular function in the patients with achalasia (Ates and Vaezi, 2015). Although the exact mechanism of the disease is not fully understood, some studies have shown evidence regarding the association of the viral, autoimmune, and neurodegenerative factors (Furuzawa-Carballeda et al., 2016; Park and Vaezi, 2005).

MicroRNAs (miRNA) are a group of small noncoding RNAs which act as gene expression regulators in different disease-related pathways (Bartel, 2004). The miRNA system involves in various physiological and pathophysiological processes and behaves as potential prognostic biomarkers (Furer et al., 2010). Several studies showed the altered miRNAs expression in various disorders, including cancers (Fang et al., 2012), immune-mediated inflammatory diseases (Singh et al., 2013; Tahamtan et al., 2016), and nervous disturbances (Wang et al., 2014a). 

Although few studies argue the association of the pathogenesis of achalasia with neurological communication, cholinergic signaling, and inflammation, studying miRNAs expression helps us to understand better the pathophysiology of achalasia. While investigating the effects of miRNAs on the pathogenesis of the abovementioned diseases has received considerable attention, their effects on the development of achalasia are still unclear. The present study performed the next-generation sequencing (NGS) with an analytical approach to identify reliable candidate miRNAs associated with the development of the disease.

## 2. Materials and methods

### 2.1. Participants and sampling

This matched case-control study was performed on 102 participants referred to the Digestive Diseases Research Center (DDRC) in the Shariati Hospital in Tehran-Iran between August 2015 and April 2016. All the patients with primary esophageal achalasia referring to the clinic for regular follow-up were recruited consecutively (N = 52). These patients aged ≥18 years old were diagnosed based on the clinical, radiological, endoscopic findings and high-resolution manometry. All the patients received the same pneumatic dilatation treatment and were classified into excellent, good, moderate, and poor categories according to the outcome. Patients in the excellent and good categories were considered as good responses to the treatment, and those in the moderate and poor categories were considered as poor responses to the treatment (Hasanzadeh et al., 2010). Controls were selected randomly from the individuals without dysphagia or esophageal lesions who visited the same clinic (N = 50). All the cases and controls were matched by age (±5 years) and sex. Participants underwent the endoscopic biopsy from the LES by an expert clinician. The samples were stored at –80 ºC for the subsequent experiments. Patients with other associated motility or nonmotility disorders, malignancy, or coagulopathy were excluded from the study. 

This study was approved by the Ethics Committee of Golestan University of Medical Sciences (Ethics Code = 31078693122415). Participation in this study was optional. Informed written consent was obtained from all of the participants and their anonymity was preserved. The test results were considered confidential and only available to the physician and the moderator of the project.

### 2.2. RNA isolation and deep sequencing

Total RNA was extracted from all the samples (52 cases and 50 controls) using the Trizol reagent according to the manufacturers’ instructions (Invitrogen, Sweden). To increase the experiment power, total RNAs were obtained from each group of patients were pooled together (mixed equally) and sent for miRNA sequencing (pooled sample 1, 2, 3, 4). For the controls, total RNAs were pooled equally, and then two pooled samples were sent for miRNA profiling (pooled sample 5, 6). In brief, each pooled sample contained 15 and 25 extracted total RNAs of the patient and the control groups respectively.

The samples were sent to BGI (Beijing Genomics Institute) for miRNA sequencing. Bioanalyzer 2100 (Agilent, Santa Clara, CA) was employed to measure the RNA Integrity Number (RIN) for each sample. The samples with RIN greater than seven were considered for sequencing. The RNA purification, library construction, and sequencing procedures were conducted by the BGI Company. Each library was single-end sequenced on an Illumina HiSeq 4000 platform. The raw miRNA-Seq data were deposited and released in the Sequence Read Archive (SRA) database, with the BioProject accession number of PRJNA616451.

### 2.3. Analysis of small RNA sequencing data (NGS data)

The FASTQC was used to perform primary quality control of the miRNA-Seq data (version 0.11.5Babraham Bioinformatics (2021). FastQC [online]. Website http://www.bioinformatics.babraham.ac.uk/projects/fastqc/ [accessed 08- 03-2016]. ). Afterward, low-quality reads and adapter sequences of raw data were trimmed by the Trimmomatic software version 0.35 (Bolger et al., 2014) (parameters of trailing 20, max info 18:0.90, and minimum length 18). Reads with the length shorter than 18 bases were discarded after quality trimming, and the remaining reads were mapped against the Rfam database (Nawrocki et al., 2014) to eliminate unwanted noncoding. RNAs (rRNAs, tRNAs, snRNAs, and snoRNA). Subsequently, the remaining reads were analyzed using the miRDeep2 software version 0.0.8 to quantify known miRNAs and predict novel miRNAs (Friedländer et al., 2011). For efficient read mapping, clean reads in each sample were collapsed into a set of unique sequences with read numbers counted. Then, the unique sequences were aligned to the Ensembl GRCH37 human genome (Ensembl Release 68) and miRNAs sequences (miRBase database, version 21) (Kozomara and Griffiths-Jones, 2013). The aligned reads were quantified using the default settings of the miRDeep2 software with only one allowed mismatch within the read. On the other hand, putative novel miRNAs were predicted using the default settings in the miRDeep2 software. The predictions by the miRDeep2 software were filtered, with a miRDeep2 score >1, the length of nucleotides ≥50, and the predicted probability of being a miRNA > 60%. The difference in miRNAs expression (fold change) was analyzed by the edgeR package (version 1.4.5) in the R software. A fold change with an adjusted p-value or false discovery rate (FDR) less than 0.05 was considered statistically significant. 

### 2.4. MiRNAs target prediction and gene enrichment analyses

Potential target genes of the differentially expressed miRNAs were predicted using the three target prediction programs, including PITA (Kertesz et al., 2007), TargetSpy (Sturm et al., 2010), and RNAhybrid (Rehmsmeier et al., 2004). If a gene was predicted by at least two programs, it was considered as a putative target. Since each program uses various algorithms to predict miRNA targets and has different levels of sensitivity and specificity, using the combination of them reduced the false positive. We applied the default parameters of each program for target prediction. The 3’UTR sequences were recovered by the Ensembl BioMart^2^Ensembl BioMart database [online]. Website http://rfam.xfam.org/ [accessed 10-04-16]. and then, were used for prediction. Finally, Gene Ontology (GO) and Kyoto Encyclopedia Genes and Genomes (Qiu, 2013) were applied to analyze the potential function and pathway of target genes.

### 2.5. Quantitative reverse transcription polymerase chain reaction (qRT-PCR) analysis

For more confirmation, the expression levels of the dysregulated candidate miRNAs were measured in the esophageal tissues of the patients with achalasia (N = 28) and control individuals (N = 32) by the ABI 7300 real-time PCR machine (Applied Biosystems, USA). The process of cDNA synthesis of miRNAs was performed by the Reverse Transcription System Kit (ZistRoyesh, Iran) with a miR-specific stem-loop primer (Mohammadi-Yeganeh et al., 2013). The SNORD47 was measured as an internal normalization control using the 2^-dct^ method. For qRT-PCR statistical analysis, differences between the two groups were tested by Student’s t-test and the Mann–Whitney U test (based on normality of data distribution) in the SPSS statistical software version 16.0. The difference with a probability value less than 0.05 was considered statistically significant.

## 3. Results 

### 3.1. Esophageal tissues of the patients with achalasia expressed MiRNA profile different from the controls

Table 1 summarizes the clinical information of the patients. As demonstrated in Table 1, there is no significant difference in age (p-value = 0.48) and sex (p-value = 0.43) between the cases and controls. The miRNA sequencing results were compared between three groups: good response group including pooled samples 1 and 2 (those with good and excellent response to the dilatation treatment), poor response group consisting of pooled samples 3 and 4 (those with moderate and poor response to the dilatation treatment), and pooled samples 5 and 6 that were merged into control group to perform the transcriptome analysis based on the clinicians’ recommendation (Table 2). It was attempted to increase the statistical power through post-processing replication for each group.

**Table 1:** Clinical data for 52 achalasia patients and 50 controls.

Characteristic	Patients †	Controls †	p-value
Mean age (SD‡ ), year	43.5 (1.6)	45.8 (1.6)	0.48
Male/female no. (% male)	31/21 (59.6)	26/24 (52)	0.43
Vantrappen classification§ExcellentGoodModeratePoor	n (%)15 (28.8)15 (28.8)12 (23.1)10 (19.2)		
Achalasia subtype¶Type 1Type 2Type 3	n (%)9 (17.3)42 (80.8)1 (1.9 )		
Mean duration (months) of symptoms (SD)	32.34 (2.06)		
Baseline symptomsDysphagiaChest painRegurgitation	n (%)43 (82.7)7 (13.5)2 (3.8)		

†Unless otherwise indicated data are expressed as number (percentage) of patients. Percentages have been rounded and might not total 100. ‡SD: Standard deviation. §Vantrappen classification: Excellent, indicates no symptoms; Good, symptoms occurring less than once a week; Moderate, symptoms occurring more than once weekly; and Poor, persistent symptoms (Vantrappen and Hellemans, 1980).¶Achalasia subtype: Type 1 (classic) with minimal contractility in the esophageal body, type 2 with intermittent periodsof panesophageal pressurization, and type 3 (spastic) with premature or spastic distal esophageal contractions (Kahrilas et al., 2015).

**Table 2:** Next-generation sequencing read counts and mapping result for individual samples.

Post processing grouping	Clinical outcome after dilatation treatment		Sample ID	Total Reads	Mapped Reads	Mapped (%)
Treat 1	Good response	Excellent	Pooled sample1	30378288	15879232	0.523
		Good	Pooled sample2	26809904	13712314	0.511
Treat 2	Poor response	Fair	Pooled sample3	29249887	14814455	0.506
		Poor	Pooled sample4	30445887	16872768	0.554
Treat 3	Without treatment / Control	Control 1	Pooled sample5	24835372	10948105	0.441
		Control 2	Pooled sample6	29473046	13101058	0.445

**Figure 1:**
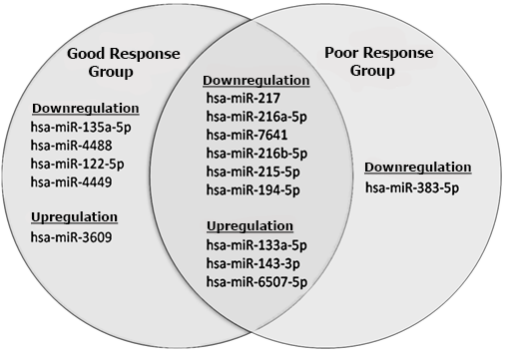
Candidate tissue miRNAs significantly differentially expressed in the patients with achalasia compared to the controls. Nine candidate miRNAs are common in samples of two achalasia groups. Good response group: achalasia patients with a good response to the pneumatic dilatation treatment. Poor response group: achalasia patients with poor response to the pneumatic dilation treatment.

The miRNA expression profiling analysis showed that 15 miRNAs were significantly differentially expressed in the tissues of the patients with achalasia (good or poor response groups) compared to the controls. Besides, our findings indicated that most of the dysregulated miRNAs (11 miRNAs) were downregulated and only four miRNAs were upregulated in the tissues of the patients with achalasia. Three miRNAs were significantly upregulated in both good and poor response group compared to the controls; miR-133a-5p (adjusted p-value < 0.001 for good response group and adjusted p-value = 0.005 for poor response group), miR-143-3p (adjusted p-value = 0.001 for good response group and adjusted p-value = 0.011 for poor response group) and miR-6507-5p (adjusted p-value = 0.001for good response group and adjusted p-value = 0.016 for poor response group). Besides, the NGS data showed hsa-miR-3609 was significantly upregulated only in a good response group compared to the controls (adjusted p-value = 0.021). Furthermore, we found six miRNAs that were downregulated significantly in both good and poor response groups (Figure1). These were miR-215-5p, miR-216a-5p, miR-216b-5p, miR-217 and miR-7641 with adjusted p-value < 0.001 and miR-194-5p (adjusted p-value = 0.01 for good response group and adjusted p-value = 0.005 for poor response group). Moreover, the good response group showed significant downregulation in the expression of four miRNAs, including hsa-miR-135a-5p, hsa-miR-4488, hsa-miR-122-5p, and hsa-miR-4449. On the other hand, the significant downregulation of hsa-miR-383-5p (adjusted p-value =0.001) was seen in the poor response achalasia group compared to the controls (Table 3). This study did not detect significant differential expression in any of the miRNAs between two groups of patients with achalasia (good and poor response groups) regarding the treatment outcome.

**Table 3:** Fifteen significant upregulated and downregulated miRNAs in the achalasia tissues (Good response group and Poor response group) versus control tissues.

MicroRNA	Treat 1				Treat 2			
	FC†	log2FC†	p-value	Adjusted p-value	FC†	log2 FC†	p- value	A p-value*
hsa-miR-217	↓0.020	–5.644	3.98E-10	2.46E-07	↓ 0.31	–1.69	9.68E-09	1.5E-06
hsa-miR-216a-5p	↓ 0.062	–4.011	1.05E-09	2.65E-07	↓ 0.047	–4.411	1.02E-10	3.14E-08
hsa-miR-7641	↓ 0.155	–2.689	1.28E-09	2.65E-07	↓ 0.160	–2.644	2.28E-09	4.71E-07
hsa-miR-216b-5p	↓ 0.08	–3.644	2.06E-09	3.18E-07	↓ 0.04	–4.644	7.52E-13	4.65E-10
hsa-miR-215-5p	↓ 0.240	–2.059	8.98E-07	0.000111	↓ 0.193	–2.373	2.39E-08	2.95E-06
hsa-miR-135a-5p	↓ 0.173	–2.531	2.14E-06	0.00022	-	-	-	-
hsa-miR-194-5p	↓ 0.368	–1.442	0.000174	0.010725	↓ 0.353	–1.502	7.62E-05	0.005888
hsa-miR-4488	↓ 0.323	–1.630	0.000571	0.029432	-	-	-	-
hsa-miR-122-5p	↓ 0.231	–2.114	0.000723	0.03438	-	-	-	-
hsa-miR-4449	↓ 0.302	–1.727	0.000835	0.036864	-	-	-	-
hsa-miR-133a-5p	↑ 35	5.129	2.89E-06	0.000255	↑ 19	4.248	7.62E-05	0.005888
hsa-miR-143-3p	↑ 6.702	2.744	1.74E-05	0.001345	↑ 5.173	2.371	0.000166	0.011374
hsa-miR-6507-5p	↑ 44	5.459	2.36E-05	0.00162	↑ 24	4.585	0.000261	0.016122
hsa-miR-3609	↑ 4.6	2.202	0.00038	0.021343	-	-	-	-
hsa-miR-383-5p	-	-	-	-	↓ 0.133	–2.910	1.28E-05	0.001317

†FC, Fold change;*A p-value§, Adjusted p-value.

### 3.2. Functional annotation of the candidate miRNAs

The biological process of GO and KEGG pathways of the 15 candidate miRNAs were analyzed based on the biological process. As detailed in Table 4, we introduced a list of the most significantly enriched terms and pathways of the target genes of candidate miRNAs involved in achalasia. Interestingly, GO analysis showed that the differentially expressed genes associated with the neuron apoptotic process (adjusted p-value = 0.004), neuronal death (adjusted p-value = 0.006), and immune response regulation (adjusted p-value = 0.008) were targeted by hsa-miR-143-3p. We found that the genes related to the cellular response to oxidative stress (adjusted p-value = 0.011), cellular aging (adjusted p-value = 0.011), axon regeneration and development (adjusted p-value = 0.031) and myelination (adjusted p-value = 0.031) were significantly enriched by the hsa-miR-217. Moreover, KEGG analysis showed that the genes involved in Glioma (adjusted p-value = 0.0001) and the Sphingolipid signaling pathway (adjusted p-value = 0.0006) were the most highly represented pathways enriched by the hsa-miR-143-3p. The genes associated with cancers, including non-small cell lung cancer (adjusted p-value = 0.001 for hsa-miR-143-3p & adjusted p-value = 0.004 for hsa-miR-217), prostate (adjusted p-value = 0.004 for hsa-miR-217), colorectal (adjusted p-value = 0.001 for hsa-miR-143-3p), bladder (adjusted p-value = 0.001 for hsa-miR-143-3p) and endometrial cancers (adjusted p-value = 0.004 for hsa-miR-217) were significantly enriched by the predicted target genes (Table 4). 

**Table 4:** The most significant enriched terms (potential function and pathway of target genes) based on biological process GO† enrichment (white rows) and KEGG‡ pathway (gray rows) of the miRNAs associated with achalasia.

miRNA	Enriched Term	Target genes	A p-value*
hsa-miR-217	Nonsmall cell lung cancer- Homo sapiens- hsa05223	E2F3;KRAS;FOX3;FHIT	0.004
	Endometrial cancer- Homo sapiens- hsa05213	TCF7L2;PTEN;KRAS;FOXO3	0.004
	Negative regulation of cell aging (GO:0090344)	PTEN;SIRT1;MARCH5	0.011
	Cellular response to oxidative stress (GO:0034599)	NR4A2;TP53INP1;FOXO3;SIRT1;HIF1A;EZH2	0.011
	Prostate cancer- Homo sapiens-hsa05215	TCF7L2;PTEN;E2F3;KRAS	0.016
	Regulation of myelination (GO:0031641)	TCF7L2;PTEN;TNFRSF21	0.041
hsa-miR-216b-5p	Melanoma- Homo sapiens- hsa05218	CDK6;CDK4;MAPK1;KRAS;FGF10	0.034
	Pathways in cancer-Homo sapiens-hsa05200	CDK6;FZD5;TPM3;CDK4;COL4A4;FZD9;TCEB2;MAPK1;KRAS;FGF10	0.036
	Signaling pathways regulating pluripotency of stem cells-Homo sapiens_hsa04550	SMAD1;FZD5;FZD9;MAPK1;LHX5;KRAS	0.036
	Nonsmall cell lung cancer-Homo sapiens-hsa05223	CDK6;CDK4;MAPK1;KRAS	0.037
hsa-miR-215-5p	Cell cycle-Homo sapiens-hsa04110	RB1;CDKN2D;CDKN2A;BUB1B;CDC7;TTK;CDC14A;ANAPC10;CDC20;ORC4;ORC1;CCNE1;RAD21;MCM3;MCM6;MAD2L1	0.012
hsa-miR-143-3p	Glioma-Homo sapiens- hsa05214	PDGFRA;MDM2;AKT1;MAPK1;BRAF;CALM3;KRAS;HRAS;IGF1R	-1.84E-05
	Sphingolipid signaling pathway- Homo sapiens- hsa04071	CERS4;SGPL1;SPTLC2;PPP2R5E;BCL2;AKT1;MAPK1;KRAS;TNF;HRAS	-6.69E-05
	MicroRNAs in cancer- Homo sapiens-has 05206	TRIM71;PDGFRA;DNMT3A;PTGS2;MAPK7;ERBB3;FSCN1;MDM2;BCL2;MAPK1;KRAS;HRAS;CD44	0.0007
	Nonsmall cell lung Cancer-Homo sapiens-hsa05223	AKT1; MAPK1; BRAF; KRAS; FHIT; HRAS	0.0012
	Colorectal Cancer-Homo sapiens-hsa05210	SMAD3; BCL2; AKT1; MAPK1; BRAF; KRAS	0.0015
	Bladder Cancer-Homo sapiens-hsa05219	MDM2; MAPK1; BRAF; KRAS; HRAS	0.0019
	Regulation of neuron death (GO:1901214)	ERBB3;UBE2V2;BCL2;AKT1;XIAP;KRAS;BRAF;HRAS;TNF;BB	0.004
	Regulation of neuron apoptotic process (GO:0043523)	ERBB3;UBE2V2;BCL2;XIAP;KRAS;BRAF;HRAS;TNF;BBC3	0.004
	Negative regulation of neuron death (GO:1901215)	ERBB3;UBE2V2;BCL2;AKT1;XIAP;KRAS;BRAF;HRAS	0.006
	Immune response regulating cell surface receptor signaling pathway GO:0002768)	PDGFRA;NCKAP1;PLEKHA1;YWHAB;LIMK1;ERBB	0.0080
hsa-miR-6507-5p	cytokinesis_(GO:0000910)	RACGAP1;PRC1;NEK7;MYH9;CEP55;RHOB	0.033
hsa-miR-135a-5p	Jak STAT signaling pathway-Homo sapiens-hsa04630	PIAS4;MYC;MPL;BCL2;STAT6;JAK2	0.002
	Signaling pathways regulating pluripotency of stem cells-Homo sapiens-hsa04550	BMPR2;APC;MYC;JAK2;SMAD5;SKIL	0.002
	Colorectal cancer-Homo sapiens-hsa05210	APC;MYC;BCL2;BIRC5	0.004
	MicroRNAs in cancer-Homo sapiens-hsa05206	MARCKS;BMPR2;APC;ROCK1;MYC;BCL2;IRS2	0.004
	TGF beta signaling pathway-Homo sapiens-hsa04350	BMPR2;ROCK1;MYC;SMAD5	0.008
	Cellular response to BMP stimulus (GO:0071773)	HEYL;GATA6;SMAD5	0.031
	Response to BMP (GO:0071772)	HEYL;GATA6;SMAD5	0.031
	neuron_projection_regeneration_(GO:0031102)	BCL2;APOA1;JAK2	0.031
	Axon development (GO:0061564)	BCL2;APOA1;JAK2	0.031
	Axon regeneration (GO:0031103)	BCL2;APOA1;JAK2	0.031
	Positive regulation of ntrinsic apoptotic signaling pathway (GO:2001244	PIAS4;SIAH1;BCL2;SKIL	0.031
hsa-miR-3609	Pathways in cancer-Homo sapiens-hsa05200	ITGB1;EGLN3;PRKCB;F2R;FZD9;XIAP;HIF1A;IGF1R;TGFBR2;BMP2;CCND1;MDM2;MAPK1;CRK;APPL1;F2RL3	0.020
	Proteoglycans in cancer Homo sapiens hsa05205	ITGB1;CCND1;PRKCB;CAV1;FZD9;MDM2;RRAS2;MAPK1;HIF1A;THBS1;IGF1R	0.020
	Endocytosis-Homo sapiens hsa04144	SH3GLB1;HSPA8;RAB5B;RAB4A;ZFYVE9;SH3KBP1;CAV1;F2R;EPS15L1;IGF1R;TGFBR2;RAB11FIP1;MDM2	0.020
	Focal adhesion- Homo sapiens-hsa04510	RAP1B;ITGB1;CCND1;PRKCB;CAV1;XIAP;PAK6;MAPK1;CRK;THBS1;IGF1R	0.020
hsa-miR-194-5p	Adherens junction- Homo sapiens-hsa04520	TJP1;EP300;RAC1;IGF1R	0.048
	Proteoglycans in cancer-Homo sapiens-hsa05205	CAV1;FZD6;RAC1;HBEGF;IGF1R	0.049
	Focal adhesion- Homo sapiens-hsa04510	CAV1;TLN2;RAC1;IGF1R;ITGA9	0.049
	HIF-1 signaling pathway-Homo sapiens-hsa04066	CDKN1B;EP300;RBX1;IGF1R	0.049

†GO, Gene Ontology; ‡KEGG, Kyoto Encyclopedia of Genes and Genomes; *A p-value, Adjusted p-value.

### 3.3. Novel predicted miRNAs in the esophageal tissue

Interestingly, the data analysis showed novel potential miRNA transcripts in the esophageal tissues were expressed in at least two different pooled samples with mean read counts greater than five in each group. All the rRNAs and tRNAs were excluded by Rfam database(Nawrocki, et al., 2014) and the identified novel miRNAs possessed the criteria of secondary structure in the RNA fold change. Thirty-six novel candidate miRNAs were identified with mammalian homologues using this approach (Table S1), but none of them was significantly changed in the achalasia. GO analysis showed that eight novel miRNAs are significantly related to the neurotransmission process (adjusted p-value = 0.03), axon development and regeneration (adjusted p-value = 0.02), cellular response to nerve growth factor (adjusted p-value = 0.03), and inflammation process (Table 5).

**Table 5:** The most significant enriched terms (potential function and pathway of target genes) based on biological process GO† enrichment of the novel candidate miRNAs in the esophageal tissues.

miRNA	Enriched Term	Target genes	A p-value*
2:46348793..46348872	Positive_regulation_of_neurotransmitter_transport_(GO:0051590)	DTNBP1	0.024
	Positive_regulation_of_neurotransmitter_secretion_(GO:0001956)		0.024
	Anterograde_axon_cargo_transport_(GO:0008089)		0.024
	Axon_cargo_transport_(GO:0008088)		0.03
	Regulation_of_neurotransmitter_secretion_(GO:0046928)		0.03
	Regulation_of_neurotransmitter_transport_(GO:0051588)		0.03
3:186787298..186787358	Neuroepithelial_cell_differentiation_(GO:0060563)	MITF	0.046
6:104646203...104646269	Cellular_response_to_interleukin-6_(GO:0071354)	GALT	0.039
	Interleukin-6-mediated_signaling_pathway_(GO:0070102)		0.027
	Response_to_interleukin-6_(GO:0070741)		0.04
7:53776229...53776317	Positive_regulation_of_interleukin8_biosynthetic_process_(GO:0045416)	PRG3	0.031
	Regulation_of_interleukin-8_production_(GO:0032677)		0.031
15:60128283...60128360	Neuron_projection_regeneration_(GO:0031102)	NEFL	0.02
	Axon_development_(GO:0061564)		0.02
	Axon_regeneration_(GO:0031103)		0.02
	Anterograde_axon_cargo_transport_(GO:0008089)		0.02
	Neurofilament_cytoskeleton_organization_(GO:0060052)		0.02
	Axon_cargo_transport_(GO:0008088)		0.02
	Response_to_axon_injury_(GO:0048678)		0.02
	Positive_regulation_of_axonogenesis_(GO:0050772)		0.024
	Negative_regulation_of_neuron_apoptotic_process_(GO:0043524)		0.036
	Regulation_of_axonogenesis_(GO:0050770)		0.036
	Negative_regulation_of_neuron_death_(GO:1901215)		0.037
	Regulation_of_neuron_apoptotic_process_(GO:0043523)		0.04
	Positive_regulation_of_neuron_differentiation_(GO:0045666)		0.044
	Regulation_of_neuron_death_(GO:1901214)		0.044
	Regulation_of_neuron_projection_development_(GO:0010975)		0.046
	Positive_regulation_of_neurogenesis_(GO:0050769)		0.047
20:38425194..38425268	Cellular_response_to_nerve_growth_factor_stimulus_(GO:1990090)	RAP1A	0.032
	Response_to_nerve_growth_factor_(GO:1990089)	RAP1A	0.032
	Positive_regulation_of_calcium_ion_transmembrane_transporter_activity_(GO:1901021)	ANK2	0.032
	Negative_regulation_of_neurotransmitter_transport_(GO:0051589)	RAP1A	0.032
	Nerve_growth_factor_signaling_pathway_(GO:0038180)	RAP1A	0.032
	Negative_regulation_of_neurotransmitter_secretion_(GO:0046929)	RAP1A	0.032
	Regulation_of_neurotransmitter_secretion_(GO:0046928)	RAP1A	0.047
6:77781479...77781527	Regulation_of_intrinsic_apoptotic_signaling_pathway_by_p53_class_mediator_(GO:1902253)	RRM2B	0.004
	Negative_regulation_of_signal_transduction_by_p53_class_mediator_(GO:1901797)		0.005
	Regulation_of_signal_transduction_by_p53_class_mediator_(GO:1901796)		0.006
	Regulation_of_intrinsic_apoptotic_signaling_pathway_(GO:2001242)		0.014
	Negative_regulation_of_apoptotic_signaling_pathway_(GO:2001234)		0.018
	Regulation_of_apoptotic_signaling_pathway_(GO:2001233)		0.028
12:29562570..29562639	Calcium-mediated_signaling_using_intracellular_calcium_source_(GO:0035584)	HOMER2	0.039
	Regulation_of_interleukin-8_biosynthetic_process_(GO:0045414)	PRG3	0.039
	Mast_cell_activation_involved_in_immune_response_(GO:0002279)	PLA2G3	0.039
	Positive_regulation_of_interleukin-8_biosynthetic_process_(GO:0045416)	PRG3	0.039
	Axoneme_assembly_(GO:0035082)	PLA2G3	0.042

### 3.4. Validation of the NGS results by the qRT-PCR analysis

Three candidate miRNAs (hsa-miR-217, hsa-miR-143-3p, and hsa-miR-133a-5p), with the highest expression changes, were selected from the NGS data to confirm the gene expression changes. The qRT-PCR was used to validate the results of NGS. The qRT-PCR findings revealed a significant decline of hsa-miR-217 expression in the achalasia tissues compared to the controls (p-value = 0.004). These findings validated the results of the same comparison conducted by the NGS method. The qRT-PCR findings of hsa-miR-143-3p and hsa-miR-133a-5p, similar to the NGS results, showed upregulated expression in the tissues of the patients with achalasia but, contrary to NGS, these findings were not significant (p-value = 0.457 and p-value = 0.840 respectively) (Figure 2).

## 4. Discussion

To the best of our knowledge, this study is the first study in which the miRNA expression in the tissues of the patients with achalasia was compared to the controls using the NGS approach. The complete pattern of the miRNAs associated with the achalasia was obtained using the NGS approach. Fifteen miRNAs had significant differential expression in the esophageal tissues of the patients with achalasia compared to the controls. It was confirmed that miR-217 was downregulated significantly, and miR-143-3p and hsa-miR-133a-5p were upregulated (p-value > 0.05) in the achalasia tissues using the stem-loop qPCR as similarly observed in the NGS results. In a recent study using the microarray method, Shoji et al. showed that only two miRNAs (miR‑361‑5p and miR‑130a) were upregulated in patients with achalasia, which is contrary to the present study. This difference may be attributed to the different methods used in each study for miRNA expression analysis. Moreover, they used middle esophageal mucosa for sampling, which could potentially have different gene expression from the LES (Shoji et al., 2017). Another study that evaluated the miRNA expression profiling by the microarray demonstrated upregulated expression of hsa-miR-133a-5p in achalasia tissue in line with our study (Palmieri et al., 2019). Both previous studies used the microarray method for sequencing. The NGS platforms have higher sensitivity and dynamic amplitude than microarrays with higher sequencing depth (Motameny et al., 2010). Furthermore, the NGS produces a more accurate and reliable sequence, even if the individual reads are less accurate (Kulski, 2016).

Functional annotation revealed that many miRNAs determined in our study are involved in neuronal cell apoptosis (hsa-miR-143-3p), myelination process (hsa-miR-217), and neuronal regeneration (hsa-miR-135a-5p). In accordance with our findings, a previous study showed the mechanism of esophageal dysfunction in response to neuronal destruction in patients with Parkinson’s disease (Qualman et al., 1984). Moreover, the current study found that hsa-miR-143-3p targeted the immune system which was shown to be dysregulated in achalasia patients. Although the etiology of primary esophageal achalasia remains unknown, several hypotheses suggest that inflammation and autoimmunity are associated with its pathogenesis (Hirano, 2006). The histopathology analysis of the esophageal tissues, indicated lymphocytic infiltration, myenteric inflammation, and aganglionosis during the achalasia (Sodikoff et al., 2016). The cytotoxic autoimmune responses can potentially trigger progressive neuronal apoptosis in the achalasia tissues (Kahrilas and Boeckxstaens, 2013). The evidence suggests that miRNAs play an important role in the development of neurodegenerative diseases (Kye and Inês do Carmo, 2014).

Some of the miRNAs that were significantly differentially expressed in the current study were previously reported as cancer-related miRNAs. For example, miR-217, assuming to have tumor suppressor function; has been reported downregulated in several cancers such as gastric cancer (Chen et al., 2015), pancreatic ductal adenocarcinoma (Vychytilova-Faltejskova et al., 2015), Esophageal Squamous Cell Carcinoma (ESCC) (Wang et al., 2015b), and colorectal cancer (Wang et al., 2015a). Moreover, similar to this study, reduced miR-216 expression was reported in other diseases, such as nonsmall cell lung cancer (Wang et al., 2014b), ESCC (Dong et al., 2016), nasopharyngeal carcinoma (Deng et al., 2011), and hepatocellular carcinoma (Liu et al., 2015). The tumor suppressor role of miR-217 and miR-216 may justify the high prevalence of esophageal cancer in patients with achalasia. Despite the pathological differences between neurodegenerative diseases (such as achalasia) and cancers, new evidence suggests that they have similar regulatory mechanisms (Grasso et al., 2014). 

The present study indicated the upregulation of hsa-miR-143-3p in the achalasia tissues of the patients. The upregulation of miR-143 in the CD4+T cells, highlights the importance of this miRNA in autoimmune diseases (Martínez-Ramos et al., 2014). This finding is in agreement with the role of autoimmunity in the formation of achalasia. 

The biological process of GO and KEGG assessments in this study demonstrated that phosphatase and tensin homolog (PTEN) and Sirtuin 1 (SIRT1) could be significant targets of miR-217 in the achalasia (Table 4). Some studies showed that PTEN has a direct role in neurodegeneration under oxidative stress conditions (Li et al., 2013b; Morris et al., 2010). SIRT1 levels are associated with neurodegenerative diseases which have a progressive and severe reduction in neuronal cells (Kim et al., 2007). These findings could be in line with the role of neurodegeneration in the development of achalasia.

Interestingly, our findings identified some genetic factors related to the candidate miRNAs similar to other studies which were associated with achalasia. For instance, the HLA genes which showed to be targeted by miR-122-5p in this study related to achalasia in another study (Ruiz-de-León et al., 2002). In the current study, some immune modulator genes, including Interleukin 10(IL-10) and Interleukin 23 Receptor (IL-23R), were predicted to be targeted by hsa-miR-143-3p and hsa-miR-216a-5 respectively (De León et al., 2010; Palmieri et al., 2016). Accordingly, these findings highlight the role of immunity and inflammation in the initiation and progression of achalasia (Table S2).

This research showed that miR-383-5p was downregulated in the patient with achalasia who had a poor response to the dilatation treatment. This miRNA might play a potential prognostic role in the prediction of the response to the treatment in patients with achalasia. However, further studies could confirm this finding. Other studies introduced the hsa-miR-383 as a tumor suppressor with a decreased level in the glioma, medulloblastoma, and testicular embryonal carcinoma cells (Li et al., 2013a; Lian et al., 2010; Xu et al., 2015; Xu et al., 2014). Our results demonstrated that dysregulated miR-216b could target the tropomyosin (TPM), the gene which encodes the beta-tropomyosin with an important role in the regulation of the calcium-dependent muscle contraction. A study showed the changes in the TPM expression on the achalasia tissues (Palmieri et al., 2016). These findings may emphasize the neuromuscular process in the pathogenesis and development of the achalasia (Table 4).

Other findings indicated that has-miR-135 was downregulated only in the patients with a good response to the treatment. Some studies showed that the induction of miR-135a expression in different types of cancers could suppress cell proliferation through target genes (c-MYC, STAT6, SMAD5, and BMPR2). Some research introduced miR-135a as a potential predictor of treatment outcome in some cancers (Yamada et al., 2013; Ahmad et al., 2018). The current study confirmed that these target genes could be considered as significant targets of has-miR-135 in achalasia (Table 4). 

This investigation found that Caveolin1 (CAV1) involving in the calcium signaling pathway could be a significant target of hsa-miR-3609 and hsa-miR-194-5p which are differentially expressed in the achalasia tissues of the patients. This finding is in line with a study that showed the CAV1 target gene was differentially expressed in the achalasia tissues with a possible function related to the achalasia pathogenesis (Palmieri et al., 2016). It is generally accepted that calcium channel blockers can support LES relaxation and esophageal peristalsis in patients with achalasia (Dughera et al., 2011). This provides further support for the role of candidate miRNAs in the etiology of achalasia.

## 5. Conclusion

In conclusion, the results of the current study provide a comprehensive analysis of miRNA expression in the achalasia and may be used as a basis for future studies to investigate the role of candidate miRNAs in the etiology of achalasia. A significant downregulation was observed in the hsa-miR-217 in the LES samples of the achalasia patients with significant enrichment in myelination process ontology. Furthermore, the NGS miRNA expression profiling might be a suitable platform to classify the achalasia into different response groups concerning the outcome of dilatation treatment.
